# A Prospective Preliminary Study Examining the Physiological Impact of Pneumatic Compression Dosing in the Treatment of Lower Extremity Lymphedema

**DOI:** 10.1089/lrb.2022.0087

**Published:** 2023-10-17

**Authors:** Vaughan Keeley, Katie Riches, Leigh Ward, Peter J. Franks

**Affiliations:** ^1^ Lymphoedema Service, University Hospitals of Derby and Burton, Derby, United Kingdom.; ^2^ School of Medicine, University of Nottingham, Nottingham, United Kingdom.; ^3^ School of Chemistry and Molecular Biosciences, The University of Queensland, Brisbane, Australia.; ^4^ Centre for Research and Implementation of Clinical Practice, London, United Kingdom.

**Keywords:** lower extremity lymphedema, pneumatic compression device dosing, Flexitouch, Perometer, bioimpedance

## Abstract

**Background:**

Optimal frequency and duration of pneumatic compression device (PCD) therapy for lymphedema is undetermined. This prospective, randomized preliminary study evaluated the impact of different PCD dosing protocols on physiological and patient-reported outcomes (PROs) to estimate treatment effects, assess the responsiveness of various measurement techniques, and identify endpoints for a definitive PCD dosing trial.

**Methods and Results:**

Twenty-one patients with lower extremity lymphedema were randomized into three groups for treatment with the Flexitouch advanced PCD: (A) once per day for 1 hour, 12 consecutive days; (B) twice per day in 1-hour treatments, 5 consecutive days; or (C) twice per day in 2-hour treatments, 5 consecutive days. Outcomes measured were changes in limb volume (LV), tissue fluid, tissue tone, and PROs. Those in group A experienced mean (standard deviation) LV reductions of 109 (58) mL (*p* = 0.003) on day 1 and of 97 (86) mL (*p* = 0.024) on day 5. Group A also showed possible single-treatment decreases in extracellular fluid volume by bioimpedance spectroscopy (BIS) on day 5. There were no consistent changes in groups B and C. Long-term assessment of LV and BIS showed no clear change. Tonometry, ultrasound, local tissue water, and PROs showed wide variation among participants.

**Conclusions:**

LV measurements showed potential benefit for 1-hour daily PCD treatment. A definitive dosing trial should include LV, BIS, and PROs in a comparison of 1- and 2-hour daily treatment protocols conducted over a study period of 4 weeks. These data may inform appropriate outcome measures for other intervention studies in lymphedema.

## Introduction

Pneumatic compression devices (PCDs) assist with limb volume (LV) control in the management of lymphedema and have been used for many years as an adjunct to home care. The Flexitouch system (FT; Tactile Medical, Minneapolis, MN, USA) simulates the low pressure, more frequent cycles of manual lymphatic drainage, a component of complete decongestive therapy (CDT).

Although several studies have reported on PCD use for treatment of lower extremity lymphedema, there is little consensus on optimal frequency and duration of treatment^1^ and no formal dosing study has been carried out. There has been a trend toward shorter treatment duration, from 8 or more hours for hospital inpatient care to 1 hour or less for current home-based care.^1^ However, it remains unclear whether 1-hour dosing is therapeutically optimal or a concession to patient convenience. The aim of this preliminary study was to evaluate the impact of various PCD dosing protocols on multiple physiological and patient-reported outcomes (PROs) to assess treatment effects and determine suitable sample size and endpoints for a definitive dosing trial.

## Methods

This three-arm, prospective, randomized, preliminary study assessed the comparative efficacy of different treatment frequency and duration in achieving a measurable effect on lower extremity lymphedema using the FT device (Supplementary Fig. S1). Many patients currently carry out a daily treatment with a PCD of 1 hour duration but it is not known whether longer durations would have a greater benefit. This study was, therefore, designed to explore whether treatments of 1 hour twice per day or 2 hours twice per day would give better results, recognizing that this may be offset by the greater time commitment required.

The outcomes were measured within day, that is, before and after treatment and before and after 5 days of treatment to determine possible long-term differences. The group which had 1 hour of treatment daily continued treatment for 12 days to explore whether this resulted in better outcomes than after 5 days. It was felt that doing this for the other groups would pose too heavy a burden and deter patients from participating in the study.

The protocol was approved by the National Research Ethics Service Committee East Midlands, Derby, United Kingdom (revised May 2014). Written informed consent was obtained from all study participants. Recruitment was conducted among patients who presented to the Derby Lymphedema Service between December 2013 and March 2019.

### Inclusion and exclusion criteria

Patients who had undergone treatment for clinically confirmed primary or secondary lower extremity lymphedema were eligible for enrollment. Participants had to be ≥18 years old and present with pitting unilateral or bilateral leg edema (International Society of Lymphology stage 2).^2^ Participants were required to be using adequate compression garment(s) as determined by the physician (minimum 20 mm Hg, ≤3 months old) and attend all in-clinic treatments.

Exclusion criteria included body mass index (BMI) >40; active or recently treated cancer; active infection or inflammation, recent venous thromboembolic disease, chronic venous insufficiency, heart failure, chronic kidney disease, poorly controlled asthma, peripheral artery disease; presence of an open wound, and pregnancy. At the screening visit, duplex ultrasound (US) tests were used to rule out significant venous insufficiency (defined by reflux >2 seconds).

It should be noted that under an earlier version of the protocol (July 2013), 468 patients were screened, leading to only one recruitment. Due to this slow recruitment, the following exclusion criteria were revised to the above: primary lymphedema, compression garment <30 mm Hg and BMI >35.

### Treatment allocation

**FIG. 1.**
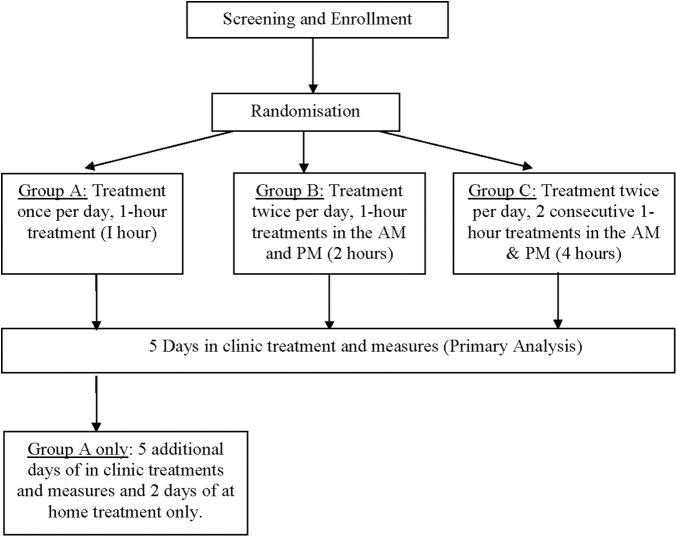
Study flow diagram.

Enrolled participants were allocated randomly and in equal numbers into one of three treatment groups by a sealed envelope method (Fig. 1). A treatment was defined as 1 hour of lower extremity FT treatment in accordance with device instructions for use, with pressure set to 55–30 mm Hg, varying from distal to proximal ends of the full limb/truncal garment set.

- Group A received one treatment per day for 12 consecutive days. The first five treatments were administered in clinic followed by two patient-administered treatments at home (Saturday and Sunday) for participant convenience, concluding with an additional five in-clinic treatments (12 total hours of treatment).
- Group B received two in-clinic 1-hour treatments per day (AM and PM with at least 2 hours between treatments) for 5 consecutive days (10 total hours of treatment).
- Group C received two consecutive 1-hour in-clinic treatments two times per day (AM and PM with at least 2 hours between treatment sessions) for 5 consecutive days (20 total hours of treatment).

In participants with bilateral lymphedema, the leg with the largest volume was studied. Treatment of contralateral legs was maintained as it had been before the study. Investigators used a pressure measuring device (PicoPress; Microlab Elettronica Sas, Padua, Italy) to ensure uniform application of the device across all participants before inflation (7–10 mm Hg).

### Endpoints

The study evaluated changes in tissue fluid, tissue tone, and PROs. Measurements of the treated and untreated limb were taken before and after each treatment, while PROs were assessed at baseline and after final treatments. A clinician assessed participants for any signs or symptoms that required medical intervention or study discontinuation.

### Fluid changes

Limb volume measurements, especially in the short term, provide a proxy measure of fluid changes. These were taken with an optoelectronic system (Perometer 400T; Pero-System Messgeräte GmbH, Wuppertal, Germany).^3,4^

Extracellular fluid (ECF) changes in the whole limb were assessed by bioimpedance spectroscopy (BIS) of the individual lower limbs,^5^ measured as changes in electrical resistance at zero frequency (R_0_) using an SFB7 impedance spectrometer (ImpediMed, Queensland, Australia) according to the protocol as described by Steele et al.^6^ Increases in R_0_ values correspond proportionally to decreases in ECF.^7^

Local tissue water (LTW) was assessed by measuring tissue dielectric constant (TDC) with a Moisture Meter D (Delfin Technologies, Kuopio, Finland), at specific anatomical sites (on dorsum of foot, medial malleolus, below patella, above patella, inguinal ligament, and lower abdominal wall) using two probes that measure LTW to depths of 2.5 and 5 mm, respectively.^8^

Ultrasound with a 5–16 MHz probe (Fujifilm Sonosite, Bothell, WA, USA), was used to measure skin thickness at the same anatomical sites as TDC. Skin thickness (epidermis and dermis) has previously been shown to be increased uniformly around the arm in breast cancer-related lymphedema and correlates strongly with the degree of swelling.^9^

### Tissue tone

A tissue tonometer (Flinders University, Adelaide, Australia) was used at the same defined locations as TDC to estimate the extent of pitting and fibrotic induration.^10^

### Patient-reported outcomes

PROs assessed symptoms and quality of life (QoL). Symptom assessment was obtained using the Measure Yourself Medical Outcome Profile (MYMOP) and QoL using a lymphedema-specific QoL questionnaire (LYMQOL). The MYMOP questionnaire was designed to be not only a patient-generated outcomes questionnaire, problem-specific, but also focused on general wellbeing.^11^ A lower score represents a better QoL. LYMQOL is a validated lymphedema QoL assessment tool that covers symptoms, body image/appearance, function, and mood, as well an overall QoL score.^12^ A lower score in the four domains represents a better QoL but for the “overall QoL” section, a higher score represents a better QoL.

### Efficacy analysis

This preliminary study was not powered for statistical analysis of differences in outcomes between the three groups. Physiological responses to both individual daily treatments and changes at the completion of the course of treatment (on day 5 for all groups) were studied to assess short- and long-term effects.

### Assessment of measurements

To evaluate the potential future use of the various methods used in this study, the dispersion of measurements and their responsiveness to change were assessed. The coefficient of variation (CV) is used as a standardized measure of data dispersion, that is, the larger the CV (%), the less consistent the data. In this study, the CV is a measure of both biological variation and the inherent error of each measurement technique. The day 1 CVs of all measurements used in this study were calculated to assess which measurements exhibited the greatest consistency and thus may be of greatest utility in a future study.

The responsiveness to change was examined a posteriori by comparing the change in each measure at the end of day 5 from baseline on day 1 with the change in LV measured by perometry in group A as the gold standard (as this demonstrated a significant reduction over this time).

### Sample size calculation

Sample size estimates for a future study were derived from a variety of scenarios assuming normally distributed data using the z-score method for independent (unmatched) samples.

### Screening log

To assess the feasibility for recruiting to a future definitive study, a screening log was maintained to record the number of patients eligible for this study and the numbers recruited.

## Results

A total of 2576 patients were screened leading to 20 recruitments (July 2014 to November 2018), with one participant having been recruited under the first version of the protocol. The original plan was to recruit 10 participants in each group but even with the amendments, recruitment was slow. Therefore, it was decided to carry out the interim analysis reported here when each intervention arm included seven completed participants.

In total, 21 participants (86% female) with chronic lower limb swelling were included in the study with 7 in each group. Individuals with primary lymphedema accounted for 81% of study participants, with 76% of participants experiencing bilateral swelling. All participants had pitting edema. None had used a PCD in the last year. All participants completed the study protocol with no adverse events.

Although there was wide variation in some of the baseline characteristics, especially lymphedema duration and age, there was no statistically significant difference in any of these between the groups (Table 1).

**Table 1.** Baseline Participant Characteristics

Characteristic	Group A,* N* = 7	Group B,* N* = 7	Group C,* N* = 7	*p*-Value*^a^*
Sex (female)	5 (71.4%)	7 (100%)	6 (85.7%)	0.31
Age (years)	65.5 (6.7)	58.1 (11.1)	50.4 (18.3)	0.12
BMI (kg/m^2^)	28.08 (7.29)	30.59 (5.12)	27.94 (5.76)	0.68
LD duration (months)	112.6 (214.8)^b^	45.2 (24.6)	92.8 (39.3)	0.59
Affected limb(s)				
Unilateral	1 (14.3%)	1 (14.3%)	3 (42.9%)	
Bilateral	6 (85.7%)	6 (85.7%)	4 (57.1%)	0.35
Trial limb				
Right	3 (42.9%)	2 (28.6%)	4 (57.1%)	0.56
Left	4 (57.1%)	5 (71.4%)	3 (42.9%)	

Results are reported as mean (SD) or *n* (%).

^a^ Pearson's chi squared used for dichotomous variables (sex, limb). ANOVA used for continuous variables (age, BMI, duration).

^b^ LD duration in group A is skewed by one participant who had their LD for 50 years (607 months).

BMI, body mass index; LD, lymphedema; SD, standard deviation.

**FIG. 2.**
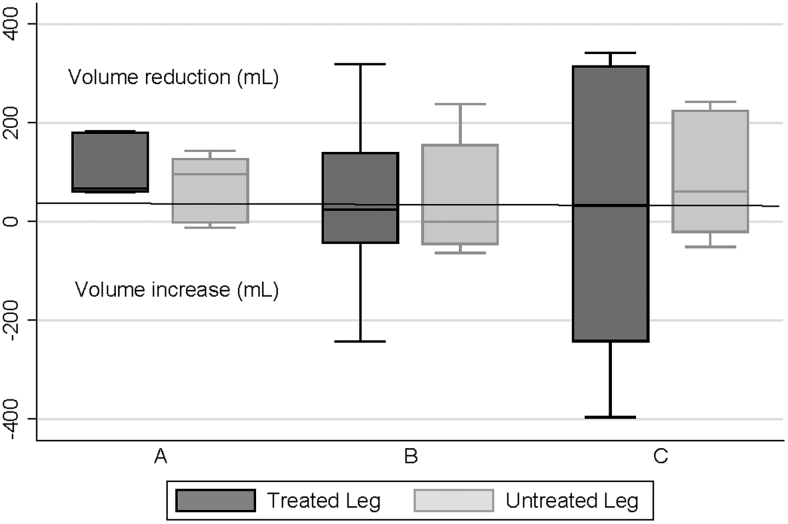
Limb volume changes (mL) after final treatment on day 1. The *line* within the box indicates the median, the upper and lower limits of the box indicate the 25% and 75% quartiles, and the *lines* extending from the box indicate the minimum and maximum values.

**FIG. 3.**
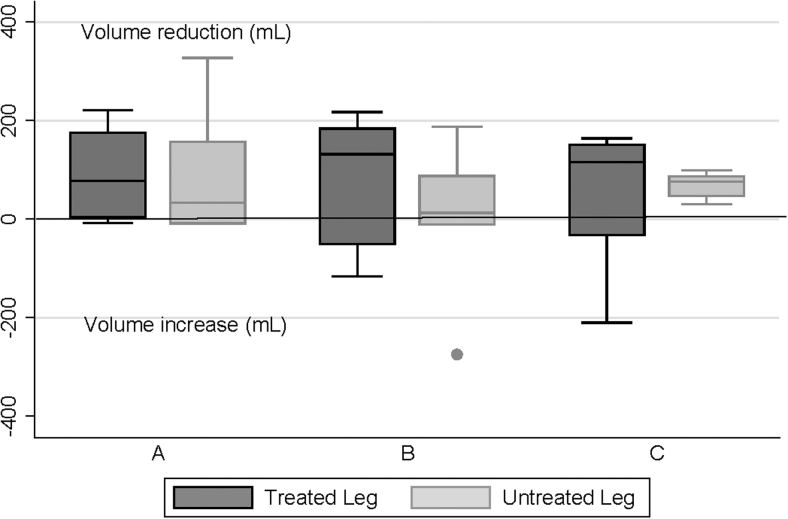
Limb volume changes (mL) after final treatment on day 5. The *line* within the box indicates the median, the upper and lower limits of the box indicate the 25% and 75% quartiles, and the *lines* extending from the box indicate the minimum and maximum values. The *bullet point* indicates an outlier in untreated limb of one participant in group B.

Figures 2 and 3 show the distribution of LV changes following final treatments on days 1 and 5, respectively. Group A experienced mean volume reductions in treated limbs with mean (standard deviation) reductions of 109 (58) mL after day 1 and 97 (86) mL after day 5 treatment sessions. Despite the small sample size, both reductions reached statistical significance (*p* = 0.0026 and *p* = 0.024, respectively). However, there were no consistent changes in groups B and C, with some participants experiencing LV increases. Interestingly, a reduction in LV was seen in most untreated limbs especially on day 5 (Fig. 3).

**FIG. 4.**
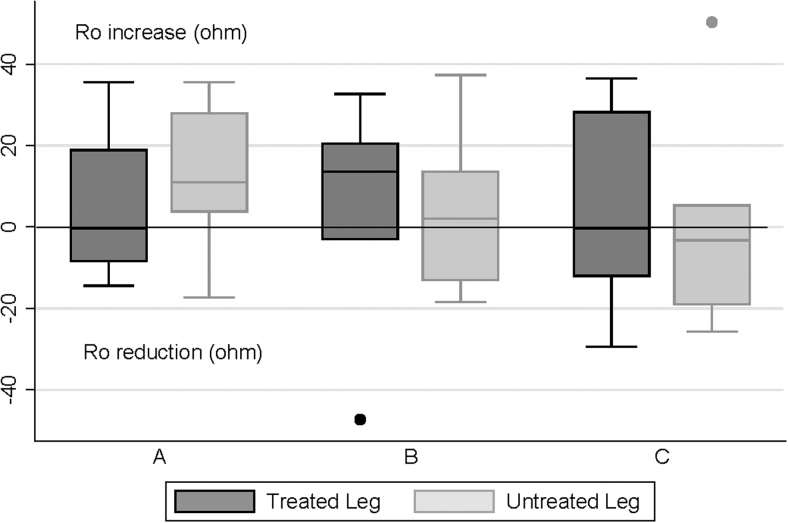
Bioimpedance changes (R_0_) after final treatment on day 1. The *line* within the box indicates the median, the upper and lower limits of the box indicate the 25% and 75% quartiles, and the *lines* extending from the box indicate the minimum and maximum values. The *bullet points* indicate outliers in treated limb of one participant in group B and untreated limb of one participant in group C.

**FIG. 5.**
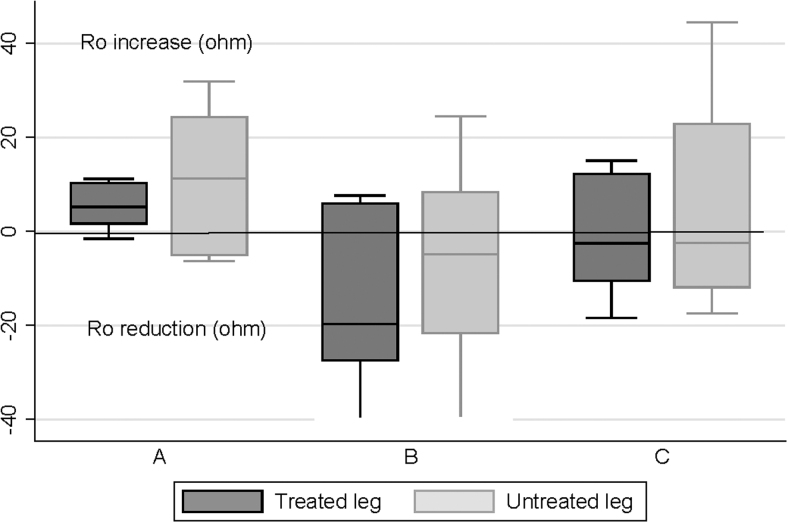
Bioimpedance changes (R_0_) after final treatment on day 5. The *line* within the box indicates the median, the upper and lower limits of the box indicate the 25% and 75% quartiles, and the *lines* extending from the box indicate the minimum and maximum values.

There were wide variations in the change in ECF as measured by BIS (R_0_) after the final treatment on day 1, with no consistent pattern evident (Fig. 4). This applied to treated and untreated limbs in all three groups. A similar pattern was seen for groups B and C on day 5, but a reduction in ECF was seen in most treated (and untreated) limbs in group A (Fig. 5).

To determine whether there were any long-term effects, baseline measures of LV and BIS (R_0_) on day 5 were compared with those on day 1 (Tables 2 and 3). These showed no consistent differences with wide variations. There were similar findings when day 12 was compared with day 1 in group A (Supplementary Tables S1 and S2).

**Table 2.** Limb Volume Measurements (mL) on Day 1 and Day 5

Day	Parameter	Group A,* N* = 7		Group B,* N* = 7		Group C,* N* = 7	
		Treated limb	Untreated limb	Treated limb	Untreated limb	Treated limb	Untreated limb
1	Baseline pretreatment LV (mL)	7729 (1472)	7328 (1954)	9209 (1818)	8705 (1429)	8976 (967)	7956 (1229)
5	Baseline pretreatment LV (mL)	7642 (1526)	7302 (2079)	9239 (1823)	8697 (8606)	9074 (993)	7946 (1155)
**Change from start of day 1 to start of day 5**							
	LV reduction (mL)^a^	87 (113)	26 (157)	−30 (121)	8 (10)	−98 (312)	11 (163)
	Percent reduction^a^	1.24 (1.54)	0.72 (1.91)	−0.34 (1.50)	0.16 (1.07)	−1.15 (3.50)	0.02 (1.85)

Results are presented as mean (SD).

^a^ A negative reduction indicates an increase in limb volume.

LV, limb volume.

**Table 3.** Bioimpedance Measurements (R_0_) (ohms) at Day 1 and Day 5

Day	Parameter	Group A,* N* = 7		Group B,* N* = 7		Group C,* N* = 7	
		Treated limb	Untreated limb	Treated limb	Untreated limb	Treated limb	Untreated limb
1	Baseline pretreatment R_0_	231 (44)	282 (88)	256 (70)	290 (78)	221 (33)	314 (91)
5	Baseline pretreatment R_0_	238 (56)	286 (84)	294 (98)	320 (85)	219 (40)	313 (95)
Change in R_0_ (day 5–day 1)							
	R_0_ change	7.0 (18.6)	4.1 (18.7)	38.2 (73.2)	29.9 (73.0)	−2.1 (25.6)	−1.0 (32.1)
	Percent change	2.1 (6.3)	1.6 (6.1)	9.3 (18.8)	7.1 (18.2)	−2.4 (13.4)	−1.1 (12.5)

Results are presented as mean (SD).

R_0_, bioimpedance.

The CV for baseline perometry and BIS measurements on day 1 were 17.8% and 21.1%, respectively.

In terms of responsiveness to change, BIS changes on day 5 in group A seemed to reflect changes in volume.

Other parameters measured (tonometry, US, TDC, MYMOP, and LYMQOL) showed no consistent pattern with wide variations in within-day changes in response to treatment, long-term effects to end of day 5, baseline CVs, and responsiveness to change (Supplementary Tables S3–S8).

Therefore, for the physical assessment methods, perometry and BIS showed the greatest consistency and responsiveness to change and, hence, utility for future studies. LYMQOL is the better PRO, based on its lower CVs (23.7%–35.5%) than those of MYMOP (Supplementary Tables S7 and S8).

Sample size calculations for a future definitive study, based on the effect sizes of LV changes from baseline day 1 to after the final treatment on day 5 in each group in this preliminary study, are provided in Supplementary Table S9. There are four proposed models in each comparison of either A versus B, A versus C, or B versus C. According to this, both A versus B and A versus C have high effect sizes, with B versus C having a low/moderate effect size. The results indicate that the percentage of nonoverlap was greatest between A and B and lowest between B and C. In summary, the optimum definitive study would be a two-group comparison of the A and B protocols, which with a power of 90% at a *p*-value of 0.05 would require a sample size of 14 participants in each group.

## Discussion

Evidence supporting at-home PCD therapy is generally based on studies involving dosages of 1 hour or less^1^; however, it remains unclear whether this 1-hour dosing standard is physiologically based. This preliminary study was designed to obtain data to inform a definitive study of whether a longer duration of treatment would be more effective. While great caution must be exercised in interpreting the results, the finding that 1 hour of daily treatment appeared to produce better within-day LV results compared with 2- and 4-hour daily treatments challenges this hypothesis (Figs. 2 and 3). Furthermore, while BIS measurements of ECF on day 1 showed a wide variation of results for all groups (Fig. 4), on day 5, most group A participants experienced ECF reductions (Fig. 5).

There have been few studies of the short-term effects of PCD therapy that apply pressure in the low range (30–60 mm Hg) recommended by international guidelines^13^; and a recent systematic review.^1^ Studies of CDT have demonstrated that most LV reduction occurs within the first day of treatment.^14^ The results of our study, in particular, the LV reduction following a single treatment, align with this pattern.

To examine whether these potential improvements immediately following a 1-hour treatment have a long-term benefit, pretreatment LV and BIS values on day 5 were compared with those on day 1 (Tables 2 and 3). There were no clear differences in any of the groups, including group A, at day 5 and day 12 (Supplementary Tables S1 and S2), suggesting that potential LV and BIS improvements immediately following treatment are not sustained. However, it is recognized that this study has a limited follow-up period and possible long-term benefits may not have been detected. A reduction of LV was shown in breast cancer-related arm lymphedema after 12 weeks of home treatment with the FT device in a previous pilot study.^15^

The apparent effect of unilateral treatment on contralateral limbs (Figs. 2–5) deserves comment. This effect has been seen in previous studies.^16^ It is suggested that the truncal component of the FT device may have facilitated contralateral limb drainage. In addition, particularly for those in groups B and C, there could have been an effect from lying supine for several hours.

In developing a future definitive study design, we considered the following several factors.

### Inclusion/exclusion criteria

The recruitment logs demonstrated that this was a difficult study to recruit to, largely because of its intensity and the time commitment required. The initial inclusion criteria were limited to secondary nonvenous lymphedema. After screening hundreds of patients without success, the inclusion criteria were broadened. Despite this, it took an additional 4 years to recruit a modest sample size of 21 participants. Allowing participants to conduct their own PCD treatments at home with fewer clinic visits for measurements should facilitate recruitment in a future trial.

With the benefit of hindsight, the decision to exclude patients with venous insufficiency seems unnecessarily limiting, as lymphedema related to venous insufficiency is common,^17^ and it is now recognized that all chronic edema stems from lymphatic dysfunction and should be considered lymphedema.^18,19^

### Sample size and study design

This study suggests that longer daily dosage may not necessarily be more effective. The sample size calculations (Supplementary Table S9) suggest that comparing group B with group C is unlikely to be productive. In addition, it is impractical for many patients to undertake 2 hours of treatment twice daily (as in group C) on a regular basis. Therefore, a study comparing group A with group B is not only the better option based on the effect size data but also should facilitate recruitment and compliance with ongoing use outside of the trial setting.

To reduce the burden of the study, most daily treatments could be carried out at home with attendance for pre- and posttreatment assessments weekly. To examine potential long-term effects, it is suggested that participants are followed up for a longer period (e.g., weekly for 1 month), although the data from this study do not inform the optimum follow-up period.

### Outcome measurement techniques

This study assessed the CV and responsiveness to change of a wide range of measurements used in previous lymphedema studies. Perometry and BIS measured both single-treatment effects and those over the 5 days of the study. While tonometry,^20^ US (of subcutaneous tissues),^21^ and TDC^22^ have contributed valuable information on edema and tissue changes/fibrosis in long-term lymphedema studies, these measures provided little added perspective on treatment effects over the 5-day study period. For a future dosing trial, it is recommended that physical measurements are limited to LV and BIS, although it is recognized that TDC can be particularly helpful in identifying local fluid changes in different regions of the limb.

PROs are also recommended but given the CVs found here, it is suggested that they are limited to LYMQOL, to reduce the burden on participants for completing questionnaires.

This preliminary study succeeded in defining a feasible framework for a future definitive trial of PCD dosing. Strengths of this study include the broad set of measurements assessed and the comparison of short- and medium-term effects. Limitations include the difficulty in recruiting participants which led to a failure to recruit the planned number; a study population unrepresentative of real-world clinical practice; and the lack of a control group of “no treatment” (as the use of PCDs was considered an established treatment).^1^

## Conclusions

In this preliminary study, perometry and BIS were shown to be the better measurements to determine the response to PCD treatment, while tonometry, US, and TDC were of lesser utility. The findings indicate a potential benefit for 1 hour of daily treatment compared with twice-daily treatments of 1 or 2 hours. A future definitive randomized controlled trial design is proposed. These data are also of wider value in informing the design of other studies examining the outcome of interventions for the treatment of lower limb lymphedema.

## Supplementary Material

Supplemental data
