# Supplementary material for: A Prospective Preliminary Study Examining the Physiological Impact of Pneumatic Compression Dosing in the Treatment of Lower Extremity Lymphedema
Source: Lymphat Res Biol. 2023 Oct 17;21(5):456–62. doi: 10.1089/lrb.2022.0087 (PMC10615048; doi:10.1089/lrb.2022.0087)
Supplement: Supplemental data [file Supp_Data.docx]

**Supplementary Materials for “A Prospective Preliminary Study Examining the Physiological Impact of Pneumatic Compression Dosing in the Treatment of Lower Extremity Lymphedema”**

Vaughan Keeley, PhD, FRCP^1,2^, Katie Riches, PhD, RN^1^, Leigh Ward, PhD^3^, Peter J. Franks, MSc, PhD^4^


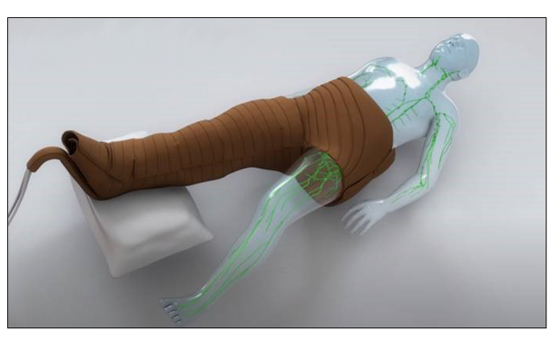


Supplementary Figure 1. The Flexitouch Advanced Pneumatic Compression Device (Tactile Medical, Minneapolis, MN) with the Lower Extremity Garment

1. **Day 12 Data for Group A**

Tables S1 and S2 present the limb volume and bioimpedance changes at day 12 for group A.

Table S1 Limb Volume Changes Day1 to Day 12 (Group A)

| **Day** | **Parameter** | **Treated Limb** |
| --- | --- | --- |
| 1 | Baseline pretreatment LV (mL) | 7729 (1472) |
| 12 | Baseline pretreatment LV (mL) | 7651 (1560)) |
|  | LV reduction (mL) | 78 (129) |
|  | Percent LV reduction | 1.18 (1.66) |

Results are presented as mean (SD).

LV, limb volume; mL, milliliter; SD, standard deviation

Table S2. Bioimpedance Measurements (ohms) Changes Day 1 to Day 12 (Group A)

| **Day** | **Parameter** | **Treated Limb** |
| --- | --- | --- |
| 1 | Baseline pretreatment R_0_ | 231 (44) |
| 12 | Baseline pretreatment R_0_ | 240 (56.6) |
| Increase in R_0_ (day12 – day1) | | |
|  | R_0_ change | 8.6 (23.7) |
|  | Percent change | 3.38 (9.11) |

Results are presented as mean (SD).

R_0_, bioimpedance; SD, standard deviation.

1. **Tonometer, Ultrasound, TDC, and PRO Data**

Tables S3 through S8 present the Day 1 and Day 5 measurements for tonometry, ultrasound, tissue dielectric constant, and PROs by treatment group.

Table S3. Tonometry Measurements (mm) at Day 1 and Day 5

| **Location** | **Period** | **Group A** | | **Group B** | | **Group C** | |
| --- | --- | --- | --- | --- | --- | --- | --- |
|  |  | **Mean** | **SD** | **Mean** | **SD** | **Mean** | **SD** |
| Dorsum of foot  (CV=51.6%) | Day 1 baseline | 1.94 | 0.87 | 2.04 | 1.69 | 2.51 | 0.58 |
|  | Day 5 end | 2.39 | 1.26 | 2.55 | 1.02 | 2.49 | 1.18 |
|  | Difference (day 1 – day 5) | -0.44 | 1.11 | -0.51 | 1.97 | -0.16 | 1.07 |
| Medial malleolus  (CV=31.3%) | Day 1 baseline | 5.35 | 1.31 | 5.37 | 2.07 | 5.44 | 1.86 |
|  | Day 5 end | 5.59 | 1.88 | 4.68 | 2.17 | 4.29 | 1.99 |
|  | Difference (day 1 – day 5) | -0.24 | 1.55 | 0.69 | 3.55 | 1.15 | 3.29 |
| Below patella  (CV=36.5%) | Day 1 baseline | 3.42 | 1.31 | 3.65 | 0.83 | 4.33 | 1.87 |
|  | Day 5 end | 2.25 | 0.69 | 3.80 | 1.47 | 5.13 | 1.59 |
|  | Difference (day 1 – day 5) | 1.18 | 1.59 | -0.15 | 1.97 | -0.79 | 1.09 |
| Above patella  (CV=18.6%) | Day 1 baseline | 6.07 | 0.95 | 6.94 | 1.68 | 6.48 | 0.82 |
|  | Day 5 end | 5.87 | 0.65 | 6.20 | 2.83 | 6.39 | 1.02 |
|  | Difference (day 1 – day 5) | 0.20 | 0.70 | 0.74 | 3.62 | 0.09 | 1.06 |
| Inguinal ligament  (CV=83.2%) | Day 1 baseline | 7.63 | 3.23 | 1.74 | 2.61 | 5.52 | 4.35 |
|  | Day 5 end | 5.94 | 3.65 | 0.83 | 0.57 | 4.69 | 3.41 |
|  | Difference (day 1 – day 5) | 1.70 | 5.89 | 0.91 | 2.97 | 0.83 | 2.14 |
| Abdominal wall  (CV=108.3%) | Day 1 baseline | 0.85 | 0.15 | 1.93 | 2.25 | 0.92 | 0.02 |
|  | Day 5 end | 2.01 | 2.96 | 1.20 | 0.48 | 1.22 | 0.46 |
|  | Difference (day 1 – day 5) | -1.16 | 2.93 | 0.72 | 2.37 | -0.30 | 0.46 |

CV% for the first measurement on day 1 for the 6 sites were in the range of 18.6% to 108.3%.

CV, coefficient of variation; SD, standard deviation.

Table S4. Ultrasound Measurements (cm) at Day 1 and Day 5

| **Location** | **Period** | **Group A** | | **Group B** | | **Group C** | |
| --- | --- | --- | --- | --- | --- | --- | --- |
|  |  | **Mean** | **SD** | **Mean** | **SD** | **Mean** | **SD** |
| Dorsum of foot  (CV=50.6%) | Day 1 baseline | 0.34 | 0.15 | 0.16 | 0.06 | 0.22 | 0.05 |
|  | Day 5 end | 0.28 | 0.06 | 0.17 | 0.09 | 0.20 | 0.05 |
|  | Difference (day 1 – day 5) | 0.06 | 0.20 | -0.02 | 0.11 | 0.02 | 0.04 |
| Medial malleolus  (CV=41.6%) | Day 1 baseline | 0.24 | 0.12 | 0.25 | 0.12 | 0.26 | 0.09 |
|  | Day 5 end | 0.27 | 0.11 | 0.18 | 0.08 | 0.31 | 0.16 |
|  | Difference (day 1 – day 5) | -0.31 | 0.07 | 0.06 | 0.16 | -0.04 | 0.12 |
| Below patella  (CV=29.6%) | Day 1 baseline | 0.28 | 0.09 | 0.23 | 0.08 | 0.29 | 0.06 |
|  | Day 5 end | 0.27 | 0.06 | 0.23 | 0.07 | 0.27 | 0.06 |
|  | Difference (day 1 – day 5) | 0.01 | 0.05 | 0.00 | 0.07 | 0.03 | 0.02 |
| Above patella  (CV=22.9%) | Day 1 baseline | 0.25 | 0.05 | 0.23 | 0.07 | 0.25 | 0.05 |
|  | Day 5 end | 0.26 | 0.05 | 0.21 | 0.04 | 0.25 | 0.06 |
|  | Difference (day 1 – day 5) | -0.01 | 0.01 | 0.01 | 0.04 | 0.00 | 0.05 |
| Inguinal ligament  (CV=27.6%) | Day 1 baseline | 0.22 | 0.07 | 0.18 | 0.06 | 0.21 | 0.03 |
|  | Day 5 end | 0.22 | 0.06 | 0.19 | 0.06 | 0.21 | 0.04 |
|  | Difference (day 1 – day 5) | 0.00 | 0.03 | 0.01 | 0.05 | 0.00 | 0.03 |
| Abdominal wall  (CV=20.4%) | Day 1 baseline | 0.21 | 0.06 | 0.20 | 0.04 | 0.22 | 0.02 |
|  | Day 5 end | 0.20 | 0.01 | 0.20 | 0.00 | 0.21 | 0.03 |
|  | Difference (day 1 – day 5) | 0.01 | 0.03 | 0.00 | 0.04 | 0.02 | 0.04 |

Coefficient of variation (CV%) for the first measurement on day 1 for the 6 sites were in the range of 20.4% to 50.6%.

CV, coefficient of variation; SD, standard deviation.

Table S5. Tissue Dielectric Constant (2.5-mm probe) Measurements at Day 1 and Day 5

| **Location** | **Period** | **Group A** | | **Group B** | | **Group C** | |
| --- | --- | --- | --- | --- | --- | --- | --- |
|  |  | **Mean** | **SD** | **Mean** | **SD** | **Mean** | **SD** |
| Dorsum of foot  (CV=14.4%) | Day 1 baseline | 46.6 | 6.8 | 45.2 | 7.6 | 44.9 | 6.1 |
|  | Day 5 end | 49.3 | 7.8 | 44.1 | 6.1 | 47.8 | 9.8 |
|  | Difference (day 1 – day 5) | -2.71 | 6.41 | 1.07 | 5.40 | -2.87 | 10.83 |
| Medial malleolus  (CV=25.9%) | Day 1 baseline | 41.2 | 10.9 | 32.5 | 7.9 | 40.9 | 9.3 |
|  | Day 5 end | 45.1 | 10.4 | 35.7 | 9.6 | 45.7 | 9.7 |
|  | Difference (day 1 – day 5) | -3.91 | 6.35 | -3.21 | 4.52 | -4.73 | 5.96 |
| Below patella  (CV=20.6%) | Day 1 baseline | 40.6 | 9.9 | 36.2 | 6.6 | 38.6 | 7.4 |
|  | Day 5 end | 45.0 | 10.5 | 36.8 | 6.4 | 40.6 | 7.7 |
|  | Difference (day 1 – day 5) | -4.44 | 5.64 | -0.61 | 5.46 | -2.00 | 5.79 |
| Above patella  (CV=16.7%) | Day 1 baseline | 30.6 | 3.7 | 29.3 | 4.0 | 28.7 | 7.0 |
|  | Day 5 end | 31.6 | 5.2 | 31.7 | 5.1 | 31.8 | 8.5 |
|  | Difference (day 1 – day 5) | -1.01 | 1.97 | -2.48 | 1.41 | -3.10 | 4.44 |
| Inguinal ligament  (CV=18.4%) | Day 1 baseline | 28.4 | 4.9 | 25.2 | 3.4 | 28.5 | 6.4 |
|  | Day 5 end | 30.0 | 6.0 | 27.2 | 3.8 | 29.4 | 8.5 |
|  | Difference (day 1 – day 5) | -1.58 | 4.10 | -2.00 | 2.05 | -0.91 | 2.95 |
| Abdominal wall  (CV=14.9%) | Day 1 baseline | 28.5 | 3.7 | 28.1 | 3.8 | 28.1 | 5.5 |
|  | Day 5 end | 28.6 | 4.0 | 29.0 | 2.8 | 29.4 | 2.8 |
|  | Difference (day 1 – day 5) | -0.07 | 4.11 | -0.94 | 1.84 | -1.32 | 5.26 |

Coefficient of variation (CV%) for the first measurement on day 1 for the 6 sites were in the range of 14.4% to 25.9%.

CV, coefficient of variation; SD, standard deviation.

Table S6. Tissue Dielectric Constant (5.0-mm probe) Measurements at Day 1 and Day 5

| **Location** | **Period** | **Group A** | | **Group B** | | **Group C** | |
| --- | --- | --- | --- | --- | --- | --- | --- |
|  |  | **Mean** | **SD** | **Mean** | **SD** | **Mean** | **SD** |
| Dorsum of foot  (CV=19.0%) | Day 1 baseline | 42.8 | 9.7 | 46.2 | 9.4 | 46.7 | 7.2 |
|  | Day 5 end | 50.1 | 4.7 | 39.9 | 14.0 | 45.8 | 13.9 |
|  | Difference (day 1 – day 5) | -7.25 | 10.88 | 6.27 | 9.58 | 0.90 | 10.38 |
| Medial malleolus  (CV=30.1%) | Day 1 baseline | 43.5 | 13.2 | 31.1 | 7.0 | 41.5 | 11.2 |
|  | Day 5 end | 43.5 | 12.9 | 34.4 | 8.5 | 44.0 | 10.4 |
|  | Difference (day 1 – day 5) | 0.00 | 1.78 | -3.35 | 9.04 | -2.45 | 6.29 |
| Below patella  (CV=25.2%) | Day 1 baseline | 37.8 | 11.2 | 33.7 | 6.5 | 39.2 | 10.0 |
|  | Day 5 end | 41.9 | 10.6 | 33.4 | 6.4 | 39.2 | 13.9 |
|  | Difference (day 1 – day 5) | -4.13 | 6.99 | 0.27 | 3.51 | -0.01 | 6.36 |
| Above patella  (CV=17.7%) | Day 1 baseline | 28.2 | 4.1 | 27.8 | 4.3 | 25.6 | 6.1 |
|  | Day 5 end | 28.4 | 4.3 | 27.6 | 4.8 | 29.0 | 8.8 |
|  | Difference (day 1 – day 5) | -0.18 | 1.33 | 0.22 | 1.75 | -3.24 | 4.57 |
| Inguinal ligament  (CV=22.8%) | Day 1 baseline | 26.3 | 4.5 | 22.8 | 3.1 | 24.7 | 8.2 |
|  | Day 5 end | 25.3 | 5.6 | 24.0 | 2.7 | 25.3 | 7.7 |
|  | Difference (day 1 – day 5) | 0.98 | 1.18 | -1.25 | 0.74 | -0.60 | 1.90 |
| Abdominal wall  (CV=14.3%) | Day 1 baseline | 25.4 | 2.8 | 26.5 | 4.0 | 24.5 | 4.3 |
|  | Day 5 end | 24.5 | 4.1 | 25.5 | 2.1 | 24.7 | 3.4 |
|  | Difference (day 1 – day 5) | 0.87 | 1.97 | 1.05 | 2.71 | -0.22 | 1.61 |

Coefficient of variation (CV%) for the first measurement on day 1 for the 6 sites were in the range of 14.3% to 30.1%.

CV, coefficient of variation; SD, standard deviation.

Table S7. LYMQOL Measurements at Day 1 and Day 5

| **Domain** | **Period** | **Group A** | | **Group B** | | **Group C** | |
| --- | --- | --- | --- | --- | --- | --- | --- |
|  |  | **Mean** | **SD** | **Mean** | **SD** | **Mean** | **SD** |
| Function  (CV=33.7%) | Day 1 baseline | 16.5 | 4.1 | 14.5 | 6.5 | 14.3 | 4.8 |
|  | Day 5 end | 16.4 | 4.8 | 13.8 | 5.3 | 14.4 | 4.3 |
|  | Difference (day 1 – day 5) | 0.58 | 1.47 | 0.64 | 2.30 | -0.05 | 2.75 |
| Appearance  (CV=34.5%) | Day 1 baseline | 16.3 | 5.4 | 13.5 | 6.0 | 14.1 | 3.8 |
|  | Day 5 end | 16.8 | 6.1 | 15.0 | 5.2 | 15.8 | 4.3 |
|  | Difference (day 1 – day 5) | -0.50 | 3.15 | -1.5 | 2.27 | -1.77 | 2.68 |
| Symptoms  (CV=35.5%) | Day 1 baseline | 10.4 | 3.7 | 10.1 | 4.1 | 8.8 | 2.9 |
|  | Day 5 end | 9.8 | 4.5 | 9.4 | 3.6 | 7.7 | 2.1 |
|  | Difference (day 1 – day 5) | 0.67 | 1.21 | 0.79 | 1.47 | 1.10 | 2.25 |
| Emotion  (CV=33.9%) | Day 1 baseline | 8.1 | 2.4 | 8.7 | 3.4 | 7.3 | 2.6 |
|  | Day 5 end | 7.8 | 1.8 | 8.1 | 1.7 | 7.6 | 3.4 |
|  | Difference (day 1 – day 5) | 0.50 | 1.76 | 0.57 | 3.55 | -0.29 | 0.95 |
| Overall QoL  (CV=23.7%) | Day 1 baseline | 7.3 | 1.3 | 6.6 | 2.1 | 8.1 | 1.6 |
|  | Day 5 end | 6.7 | 1.4 | 7.3 | 1.6 | 7.9 | 1.3 |
|  | Difference (day 1 – day 5) | -0.33 | 0.82 | 0.71 | 1.50 | -0.29 | 0.76 |

Coefficient of variation (CV%) for the first measurement on day 1 varied between 23.7% and 35.5%.

CV, coefficient of variation; LYMQOL, Lymphoedema Quality of Life questionnaire; SD, standard deviation.

Table S8. MYMOP Measurements at Day 1 and Day 5

| **Domain** | **Period** | **Group A** | | **Group B** | | **Group C** | |
| --- | --- | --- | --- | --- | --- | --- | --- |
|  |  | **Mean** | **SD** | **Mean** | **SD** | **Mean** | **SD** |
| First symptom  (CV=50.9%) | Day 1 baseline | 3.5 | 1.8 | 3.3 | 1.8 | 3.0 | 1.7 |
|  | Day 5 end | 3.0 | 1.4 | 2.7 | 2.0 | 2.5 | 1.9 |
|  | Difference (day 1 – day 5) | 0.50 | 0.84 | 0.67 | 0.82 | 0.25 | 1.26 |
| Second symptom  (CV=46.5%) | Day 1 baseline | 2.3 | 1.2 | 4.0 | 1.7 | 5.0 | 1.4 |
|  | Day 5 end | 1.8 | 1.0 | 4.0 | 1.7 | 3.5 | 0.7 |
|  | Difference (day 1 – day 5) | 0.33 | 0.58 | 0.00 | 0.0 | 1.50 | 0.71 |
| Activity  (CV=41.3%) | Day 1 baseline | 2.8 | 1.6 | 4.0 | 1.2 | 4.0 | 1.4 |
|  | Day 5 end | 2.2 | 0.8 | 2.6 | 2.1 | 2.3 | 2.5 |
|  | Difference (day 1 – day 5) | 0.60 | 0.89 | 1.00 | 1.73 | 0.50 | 0.71 |
| Well being  (CV=79.4%) | Day 1 baseline | 2.0 | 1.0 | 2.0 | 1.3 | 1.6 | 2.3 |
|  | Day 5 end | 1.2 | 0.8 | 1.7 | 1.1 | 1.5 | 2.1 |
|  | Difference (day 1 – day 5) | 0.6 | 0.55 | 0.29 | 1.80 | 0.0 | 0.0 |

CV, coefficient of variation; MYMOP, Measure Yourself Medical Outcome Profile; SD, standard deviation.

1. **Evaluation of Measurements for Use in Future Studies.**

To evaluate the potential future use of the various methods employed in this study, the dispersion of measurements and their sensitivity to change was assessed (see Tables S3-S6).

1. Coefficient of variation (CV)

The coefficient of variation (CV%) gives a measure of dispersion and was used on the first visit prior to the intervention. The CV% for both perometry and bioimpedance were 17.8% and 21.1%, respectively. For tonometry, the CV% varied from 18.6% to 108.3%, according to site. For ultrasound, the CV% varied between 22.9% and 50.6%. The dielectric constant performed better with CV% ranging from 14.4% to 25.9% using the 2.5-mm probe and 14.3% to 30.1% with the 5-mm probe.

1. Responsiveness to change

The responsiveness to change was examined in relation to the outcome after 5 days using limb volume measured using perometry as the gold standard in group A, which demonstrated a significant reduction.

For tonometry, 3 sites produced positive changes and 3 negative changes. The changes were large but went in opposite directions.

For ultrasound, 4 sites produced positive changes and 2 negative changes. In all but 2 the changes were minimal, and of those that were large, 1 was positive and the other was negative.

For TDC (2.5-mm probe), most changes were reasonably large, and all showed an increase in measure. An increase in TDC represents an increase in local tissue water. In addition, the changes in group A were generally similar in magnitude to the other treatment groups, which was not apparent when using perometry.

Using the 5-mm probe, there were 3 negative changes and 3 positive changes, with inconsistencies across the groups.

From this we can conclude that whilst tonometry, ultrasound, and dielectric constant may have value in examining local changes on the limb, there was insufficient evidence to support their use when examining the whole limb.

1. **Sample Size Calculations Based on Results From the Flexdose Trial**

The sample size calculations are based on the means and standard deviations of LV changes in the groups from the start of day 1 to the end of day 5. There are 4 proposed models in each comparison of either A versus B, A versus C, or B versus C.

Cohens d statistic indicates the effect size. According to this, both A versus B and A versus C have high effect sizes, with B versus C having a low/moderate effect size. The results indicate that the percentage of nonoverlap was greatest between A and B and lowest between B and C.

Table S9 Sample Size Calculations for Future Dosing Study

| **Mean (SD)^a^** | **Mean (SD)^a^** | **Effect Size (Cohens)^b^** | **Power** | **Significance** | **Sample Size per Group (Total)** |
| --- | --- | --- | --- | --- | --- |
| **Group A** | **Group B** | **1.256**  (65% nonoverlap) |  |  |  |
| 184 (60) | 60 (126) |  | 0.8 | 0.05 | 10 (20) |
| 184 (60) | 60 (126) |  | 0.9 | 0.05 | 14 (28) |
| 184 (60) | 60 (126) |  | 0.8 | 0.01 | 15 (30) |
| 184 (60) | 60 (126) |  | 0.9 | 0.01 | 19 (38) |
| **Group A** | **Group C** | **0.840**  (50% nonoverlap) |  |  |  |
| 184 (60) | -45 (381) |  | 0.8 | 0.05 | 23 (46) |
| 184 (60) | -45 (381) |  | 0.9 | 0.05 | 30 (60) |
| 184 (60) | -45 (381) |  | 0.8 | 0.01 | 34 (68) |
| 184 (60) | -45 (381) |  | 0.9 | 0.01 | 43 (86) |
| **Group B** | **Group C** | **0.370**  (25% nonoverlap) |  |  |  |
| 60 (126) | -45 (381) |  | 0.8 | 0.05 | 115 (230) |
| 60 (126) | -45 (381) |  | 0.9 | 0.05 | 154 (308) |
| 60 (126) | -45 (381) |  | 0.8 | 0.01 | 171 (342) |
| 60 (126) | -45 (381) |  | 0.9 | 0.01 | 218 (436) |

^a^ The sample size calculations are based on the means and standard deviations of LV changes in the groups from the start of day 1 to the end of day 5.

^b^ Cohens d statistic indicates the effect size. According to this, both A versus B and A versus C have high effect sizes (d≥0.8), with B versus C having a low/moderate effect size (d=0.2 to 0.5).
